# Dietary Index for Gut Microbiota and Leisure Time Physical Activity: The Potential Combined Protective Impact on Hypertension Risk

**DOI:** 10.1002/fsn3.71245

**Published:** 2025-11-27

**Authors:** Jiaxin Wang, Xinyi Lu, Yongting Zhao, Zewei Jiang, Jinghao Wang, Zhiguo Wang, Xiaofang Zhang

**Affiliations:** ^1^ Academician Collaborative Laboratory for Basic Research and Translation of Chronic Diseases, Central Laboratories The First Affiliated Hospital of Jinan University, Jinan University Guangzhou China; ^2^ Guangdong Provincial Key Laboratory for Chronic Disease Interactive Network Research Jinan University Guangzhou China; ^3^ Department of Pharmacy The First Affiliated Hospital of Jinan University Guangzhou China; ^4^ Department of Endocrinology and Metabolism The First Affiliated Hospital of Jinan University Guangzhou China; ^5^ Department of Pulmonary and Critical Care Medicine The First Affiliated Hospital of Jinan University Guangzhou China

**Keywords:** combined effects, dietary index for gut microbiota, hypertension, leisure‐time physical activity, NHANES

## Abstract

Emerging evidence highlights the gut microbiome's role in hypertension via microbial metabolites and endothelial dysfunction, while the Dietary Index for Gut Microbiota (DI‐GM) quantifies diet quality for microbiota health. In addition, leisure‐time physical activity (LTPA) also reduces blood pressure, but their combined impact on population‐level hypertension remains unclear. Therefore, this study explores the individual and joint effects of DI‐GM and LTPA on hypertension risk. To address this objective, we conducted a cross‐sectional study analysis of data from 27,643 adults in the National Health and Nutrition Examination Survey (NHANES 2007–2020). Excluding individuals with incomplete data, key variables included the DI‐GM and LTPA pattern (categorized by intensity, frequency, and regularity; regularly active defined as > 2 days/week). Weighted logistic regression models and restricted cubic splines (RCS) evaluated independent, joint, and non‐linear associations of DI‐GM and LTPA with hypertension, adjusting for covariates. The results showed that higher DI‐GM scores and greater weekly LTPA were inversely correlated with hypertension risk, exhibiting dose‐dependent patterns (16% lower odds for DI‐GM ≥ 6 vs. lowest groups; 16%–24% lower odds for LTPA (≥ 150 min/week) vs. lowest groups). RCS analysis showed a linear inverse dose–response relationship between DI‐GM and hypertension. The regularly active LTPA pattern (> 2 days/week) was linked to a 24% lower risk of hypertension (OR = 0.76, 95% CI = 0.69–0.84), and there was also a significant association observed for the weekend warrior LTPA pattern (1–2 days/week) (OR = 0.80, 95% CI = 0.64–0.99). Notably, joint analysis demonstrated that individuals with DI‐GM > 4 scores and LTPA ≥ 150 min/week or regularly LTPA pattern exhibited the lowest hypertension odds. In conclusion, both a high DI‐GM score (≥ 6 points) and sufficient LTPA (≥ 150 min/week) were independently associated with reduced odds of hypertension, with their combined effect amplifying protective benefits. Prospective studies are warranted to confirm temporality and causal pathways.

## Graphical abstract

Joint associations of dietary index for gut microbiota and leisure‐time physical activity with hypertension risk in U.S. adults.

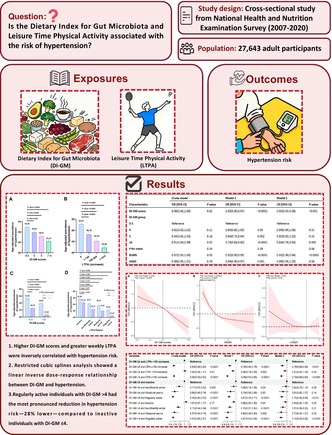

**Abbreviations**
BGMS: Beneficial gut microbiota score
CIs: Confidence intervals
CVD: Cardiovascular diseases
DASH: Dietary Approaches to Stop Hypertension
DI‐GM: Dietary Index for Gut Microbiota
DM: Diabetes
eGFR: Estimated glomerular filtration rate
FFQs: Food frequency questionnaires
FPIR: Family income‐to‐poverty ratio
HbA1c: Hemoglobin A1C
LTPA: Leisure‐time physical activity
MASLD: Metabolic dysfunction‐associated steatotic liver diseases
MET: Metabolic equivalent
MPA: Moderate PA
NCHS: National Center for Health Statistics
NHANES: National Health and Nutrition Examination Survey
OGTT: Oral glucose tolerance test
ORs: Odd ratios
PA: Physical activity
RAAS: Renin‐angiotensin‐aldosterone system
RAS: Renin‐angiotensin system
RCS: Restricted cubic splines
SCFAs: Short‐chain fatty acids
SE: Standard error
SHR: Spontaneously hypertensive rats
UGMS: Unfavorable gut microbiota score
VPA: Vigorous PA

## 1 Introduction

In recent years, the gut microbiome has emerged as a pivotal interface between dietary exposures and metabolic pathophysiology, with its dysregulation implicated in the global burden of cardiovascular‐metabolic disorders, attracting considerable scientific attention. Notably, the Dietary Index for Gut Microbiota (DI‐GM), an emerging dual‐dimensional assessment tool that integrates diet and microbiota, quantifies the regulatory potential of dietary patterns on gut microbial function with precision. Derived from extensive literature reviews and analyses of dietary components, DI‐GM resolves the biological ambiguity inherent in traditional dietary assessments, such as food frequency questionnaires (FFQs), by providing a more precise and scientifically grounded approach (Kase et al. 2024). Emerging evidence has linked the DI‐GM to cardiovascular and metabolic disease risks, encompassing conditions such as diabetes, chronic kidney disease, and metabolic dysfunction‐associated steatotic liver diseases (MASLD) (An et al. 2024; Zheng, Qi, et al. 2025; Huang et al. 2025; Zheng, Hou, et al. 2025). These associations provide critical insights into disease mechanisms and highlight potential therapeutic targets for intervention.

Hypertension, a prevalent metabolic disorder, affects approximately one‐third of the global adult population and serves as a central risk factor for cardiovascular diseases (CVD) (Carey et al. 2022). Accumulating evidence from studies has highlighted the critical role of gut microbiota dysbiosis in blood pressure regulation. Specifically, diet‐induced gut dysbiosis contributes to hypertension pathogenesis through mechanisms characterized by reduced production of short‐chain fatty acids (SCFAs) and over‐proliferation of pro‐inflammatory microbial taxa (Luo et al. 2023; Chen et al. 2019). Notably, fecal microbiota transplantation experiments have demonstrated the transmissible nature of blood pressure phenotypes (Li et al. 2017), while probiotic interventions show promise in ameliorating hypertension (Hadi et al. 2022).

In addition to dietary modifications, physical activity serves as a critical non‐pharmacological intervention for hypertension management. It is extensively supported by global guidelines due to its efficacy, safety, and cost‐effectiveness (Mancia et al. 2023; McEvoy et al. 2024). Specifically, leisure‐time physical activity (LTPA) has been consistently validated as a key lifestyle intervention for hypertension prevention and control (Ding and Xu 2023; Shariful Islam et al. 2023). Its anti‐hypertensive mechanisms include improvements in vascular endothelial function, reductions in systemic inflammation, and enhancements in antioxidant capacity (Song et al. 2022). However, there is significant individual heterogeneity in the blood pressure‐lowering efficacy of LTPA, which may be attributed to its modulator effects on the gut microbiota.

Growing evidence indicates that exercise training induces beneficial shifts in gut microbial composition, promoting the proliferation of commensal bacteria while inhibiting pathogenic taxa, which consequently enhances gut health and metabolic efficiency (Motiani et al. 2020; Yang et al. 2021; Barton et al. 2018). Recent research by Pu Y et al. further demonstrates that exercise enhances both the diversity and richness of gut microbial composition in spontaneously hypertensive rats (SHR) (Li et al. 2025), thereby promoting blood pressure regulation via microbiota‐mediated mechanisms. These findings highlight the potential joint effects between LTPA, gut microbiota dynamics, and hypertension management.

Although numerous studies have explored the impact of diet‐gut microbiota and physical activity on hypertension from the perspective of molecular mechanisms, the combined effect of these factors in the real world has not been fully studied. To date, no research has comprehensively examined the independent and joint effects of DI‐GM and LTPA on hypertension. Therefore, using data from adults of the National Health and Nutrition Examination Survey (NHANES), this study aims to investigate the combined association of diet‐gut microbiota and LTPA with hypertension prevalence.

## 2 Method

### 2.1 Study Participants

**FIGURE 1:**
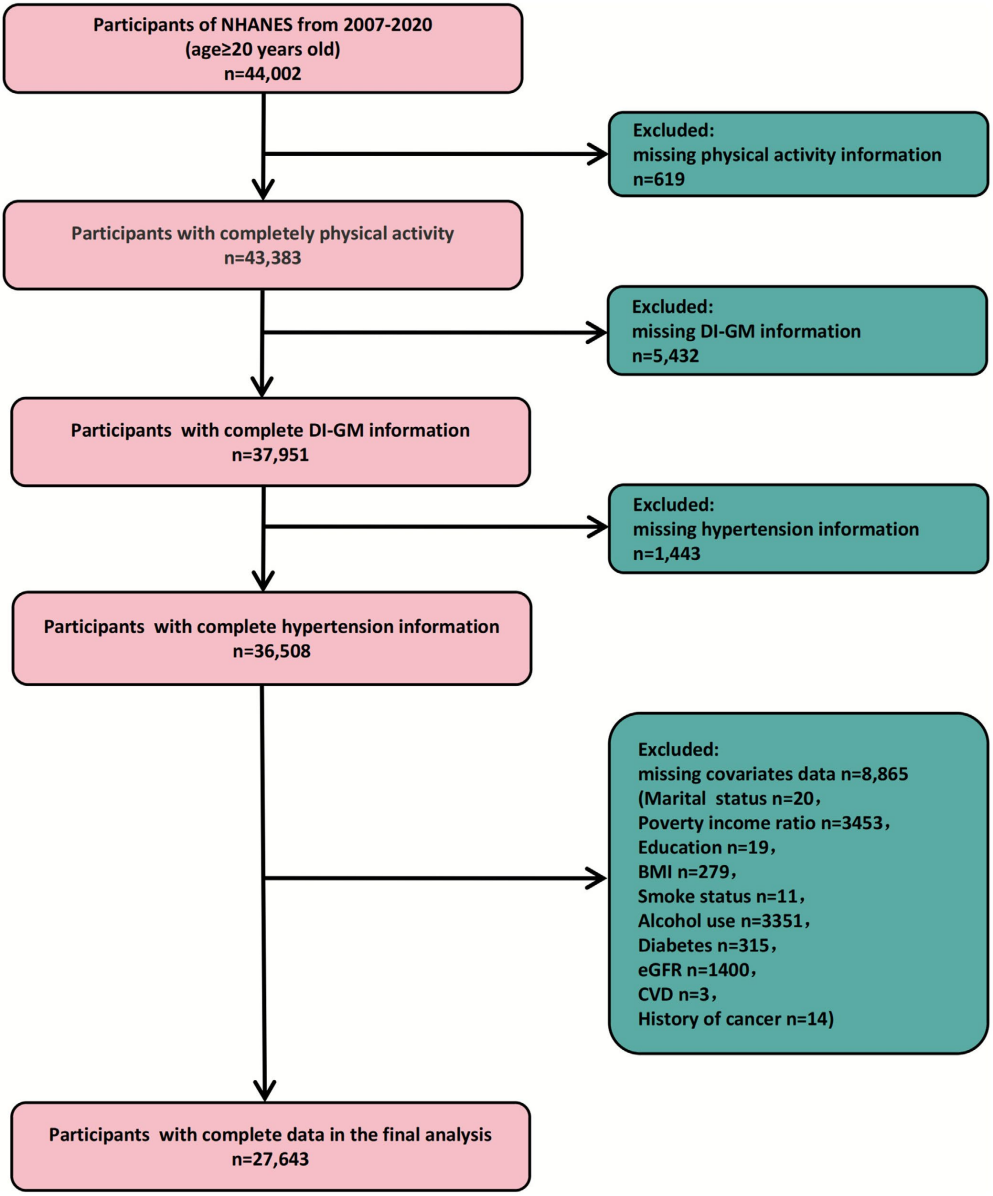
Flow chart.

The data for this study were extracted from the NHANES, a nationally representative cross‐sectional survey designed and executed by the National Center for Health Statistics (NCHS) to assess the nutritional and health status of the U.S. adult population. Since its inception in 1960, NHANES has consistently provided comprehensive information on population health, nutrition, diseases, and risk factors, utilizing standardized anthropometry measurements, laboratory tests, and in‐home questionnaire data collected from a representative sample of the individuals. The survey employs complex stratified multistage probability sampling techniques to biennially update its data which are publicly accessible. This study utilized data from a sample of adults aged 20 and older from seven cycles spanning the years 2007 to 2020. During the sample selection process, the following criteria were applied to exclude participants: (1) participants missing data on physical activity, DI‐GM scores, and hypertension information; (2) participants lacking information on demographic characteristics, lifestyle, and comorbidity covariates information. Finally, a total of 27,643 individuals were included in the final analysis. For more information on the screening process, please refer to the flowchart (Figure 1).

### 2.2 Definition of Hypertension

Blood pressure measurements were conducted by trained staff, who required participants to rest for 5 min, used a cuff covering 80% arm circumference, measured both arms, and diagnosed hypertension after three elevated readings. Participants were classified as hypertensive if they met ≥ 1 of the following criteria: (1) Prior hypertension diagnosis by a physician or healthcare provider; (2) Systolic blood pressure ≥ 140 mmHg and/or diastolic blood pressure ≥ 90 mmHg; (3) Current anti‐hypertensive medication use (Zhang, Wei, et al. 2024).

### 2.3 Definition of DI‐GM

A review of 106 eligible studies (102 intervention studies and 4 longitudinal observational studies) identified 14 foods or nutrients as components of DI‐GM. The DI‐GM components were defined based on the framework established by Kase et al. (Kase et al. 2024), encompassing 14 dietary items categorized as follows: 10 beneficial components: avocado, broccoli, chickpeas, coffee, cranberries, fermented dairy products, dietary fiber, green tea, soy products, and whole grains; 4 detrimental components: red meat, processed meats, refined grains, and high‐fat diets (defined as ≥ 40% of total energy derived from fat).

Scoring was based on gender‐specific median intake levels: for beneficial gut microbiota score (BGMS) participants received 1 point if their intake met or exceeded the gender‐specific median; otherwise, 0 points. And for unfavorable gut microbiota score (UGMS), participants received 0 points if intake met or exceeded the gender‐specific median (or ≥ 40% energy from fat for high‐fat diets); otherwise, 1 point. The DI‐GM score was calculated by summing all components. Scores were categorized into four groups: 0–3, 4, 5, and ≥ 6 for analysis (Zhang, Yang, et al. 2024; Liu and Huang 2025). We also used the weighted median of DI‐GM to determine the cut‐off values (≤ 4 and > 4) for dividing DI‐GM into two groups for sensitivity analysis.

### 2.4 Definition of LTPA

The Global Physical Activity Questionnaire was used to collect physical activity data (PA), which was then analyzed based on the World Health Organization's guidelines (Piercy et al. 2018). PA was assessed across three domains of daily activity: occupation‐related PA (OPA), transportation‐related PA (TPA), and leisure‐time PA (LTPA). Total PA was defined as the sum of these three domains. The metabolic equivalent (MET) of PA is calculated using a formula that considers the types, frequency, and duration of activities within different work areas, transportation, and leisure time. The total minutes of OPA and LTPA were twice the vigorous PA (VPA) plus moderate PA (MPA) minutes.

For LTPA, participants were classified based on whether they meet the 2018 PA guideline of 150 min of MPA per week or 75 min of VPA per week; otherwise, they do not meet the recommended standard. To evaluate the dose–response relationships between LTPA and the risk of hypertension, we also divided total LTPA into four groups: (1) 0 min/week, (2) 1–149 min/week, (3) 150–299 min/week, and (4) ≥ 300 min/week.

Additionally, based on the intensity and frequency of LTPA, we categorized participants into four groups: (1) inactive (total LTPA = 0 min/week of LTPA was reported), (2) insufficiently active (total LTPA < 150 min/week), (3) weekend warrior (total LTPA ≥ 150 min/week, exercise only 1–2 times a week), (4) regularly active (total LTPA ≥ 150 min/week, exercise more than 3 times a week) (Khurshid et al. 2023).

### 2.5 Definition of Covariates

In this study, we compiled a comprehensive set of covariate variables. Demographic variables include age, sex (female, male), and race/ethnicity (non‐Hispanic white, non‐Hispanic black, Mexican American, others). The lifestyle behavior variables include smoking status, alcohol consumption, and BMI. Smoking status (never/former, current) was included in lifestyle variables (Ren et al. 2023). The participants' alcohol consumption was assessed through a self‐reported questionnaire, with a specific focus on both lifetime and current use (within the past 12 months). Participants were classified as never/former (had < 12 drinks in their life, or did not consume alcohol last year but had a history of consuming ≥ 12 drinks within one year or throughout their lifetime) and current (Wei et al. 2024). Estimated glomerular filtration rate (eGFR) was calculated using the Chronic Kidney Disease Epidemiology Collaboration algorithm. Family income was divided by family size for a given poverty level to determine the family income‐to‐poverty ratio (FPIR), which was classified as ≤ 1.30, 1.30–3.50, and > 3.50. Participants provided responses regarding their marital status, which included being married and living with a partner, or other. Educational level was divided into two levels: high school/equivalent education or above and other. Participants with diabetes (DM) who meet any of the following criteria: (1) had been diagnosed with diabetes by a doctor; (2) had a hemoglobin A1C (HbA1c) level higher than 6.5%; (3) had a fasting blood glucose ≥ 7.00 mmol/L; (4) had a random blood glucose ≥ 11.10 mmol/L; (5) had a two‐hour oral glucose tolerance test (OGTT) ≥ 11.10 mmol/L; (6) currently using any medication or insulin to treat diabetes. CVD refers to a spectrum of disorders affecting the heart and vasculature, including coronary artery disease, angina, congestive heart failure, myocardial infarction, and stroke.

Participants were asked, “Has a doctor or healthcare provider ever diagnosed you with cancer or any malignant tumor?” Those answering “yes” were categorized as having a history of cancer. Total daily energy intake, derived from 24‐h dietary recall data, was adjusted in all model 2 to account for confounding variables and reduce extraneous variability in dietary assessments.

### 2.6 Statistical Analysis

Following the analysis recommendations of the NHANES database, this study considered the complex sample design and used the recommended weights from the NHANES database to enhance the representativeness of the data and ensure its applicability to the entire US population. We quantified the degree of multicollinearity by calculating the Variance Inflation Factor (VIF) for the main variables in the model, presented as GVIF1/(2Df). The results showed that the VIF values of all variables were at low levels (Figure S1). After gathering and organizing the data, we summarized baseline characteristics as frequencies and proportions for categorical variables and as means ± standard error (SE) for continuous variables. We constructed weighted logistic regression models to estimate independent and joint effects of DI‐GM and LTPA on hypertension. The effects were represented separately by odds ratios (ORs) and 95% confidence intervals (CIs). Three statistical models were constructed to examine the study outcomes: crude model (unadjusted), model 1 (adjusted for age, sex, and race), and model 2 (with additional adjustments including marital status, education level, PIR, BMI, smoking status, alcohol use, CVD, DM, eGFR, history of cancer, and energy intake). Restricted cubic splines (RCS) were utilized to visualize the relationships among DI‐GM, BGMS, UGMS, LTPA and hypertension. We selected 3 knots, and the positions of inflection points were marked in the nonlinear RCS plots. Finally, to ensure the stability and robustness of the results, we conducted subgroup analyses across different populations to examine the associations of DI‐GM and hypertension and performed interaction tests. Additionally, to mitigate the impact of missing variables on the results, multiple imputation via the MICE package was used to impute missing values. Ultimately, 5 imputed datasets were generated based on the variables in the statistical model. We pooled the results in accordance with Rubin's Rules to ensure the robustness and reliability of the conclusions (no weighting).

All analyses were conducted using R software version 4.4.2, and statistical significance was defined as a two‐tailed *p*‐value of less than 0.05; the data cleaning and definition for NHANES have been uploaded to a public repository (https://figshare.com/articles/dataset/_b_Data_Cleaning_and_Definition_of_NHANES_b_/30272062).

## 3 Result

### 3.1 Baseline Characteristics of Participants

**FIGURE 2:**
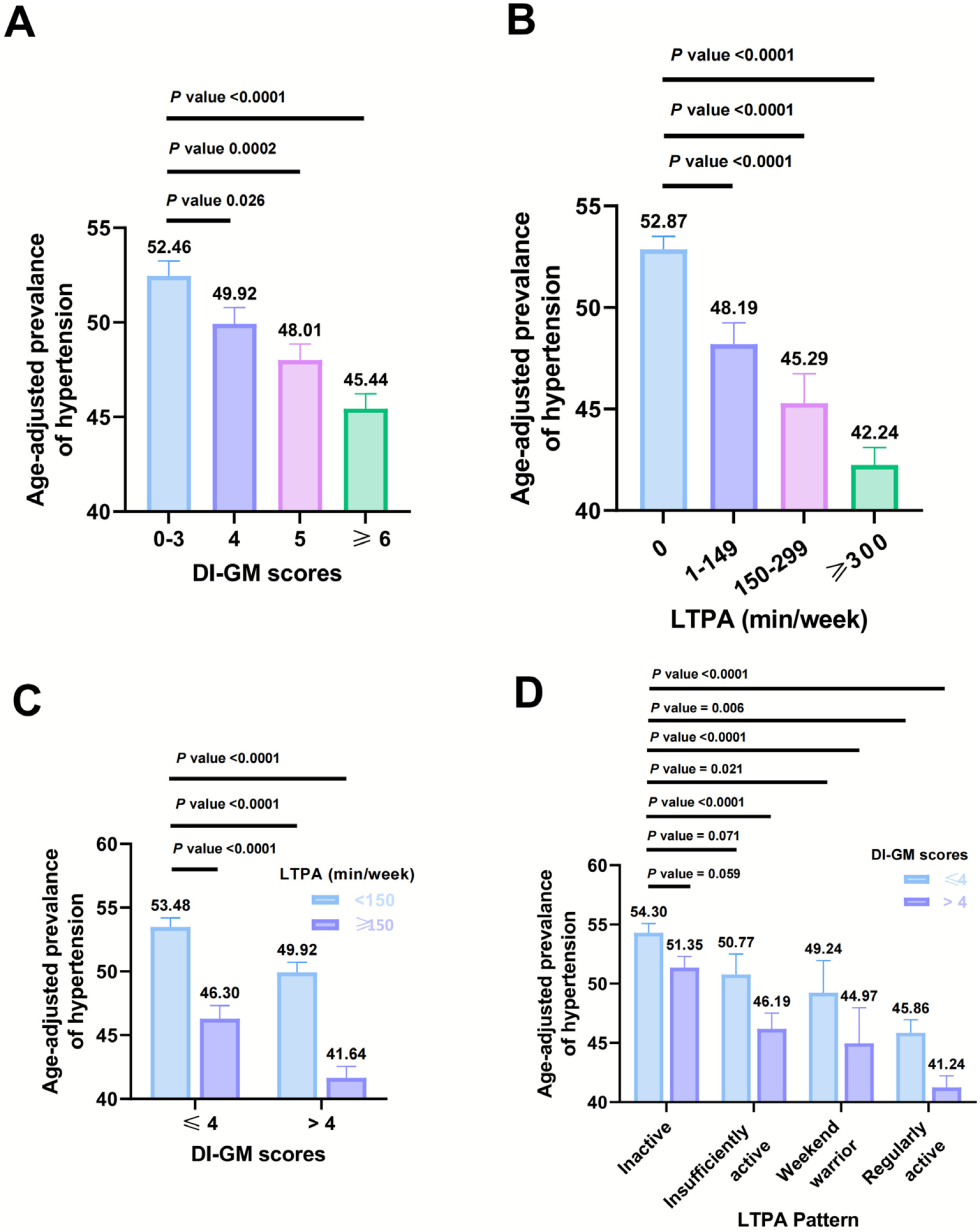
Age‐adjusted prevalence of hypertension. (A) Age‐adjusted prevelence of hypertension by DI‐GM score groups. (B) Age‐adjusted prevelence of hypertension by LTPA time. (C) Age‐adjusted prevelence of hypertension by DI‐GM score groups and LTPA time. (D) Age‐adjusted prevelence of hypertension by DI‐GM score groups and LTPA pattern. The numbers on the columns represent the prevalence rate, and the error bars represent the standard error.

After rigorous screening, this study included 27,643 participants aged ≥ 20 years from the NHANES database spanning 2007 to 2020. The characteristics of the study population are summarized in Table 1, categorized according to the DI‐GM scores. The mean age of the participants was 47.29 ± 0.26 years, including 13,835 (50.64%) female and 13,808 (49.36%) being male. Except for alcohol use, hypertension and CVD, significant differences were observed among the DI‐GM scores for all other variables. As shown in Figure 2, the age‐adjusted prevalence of hypertension significantly decreases with higher DI‐GM scores and increased leisure‐time physical activity (LTPA) (Figure 2A,B). Among participants stratified by combinations of LTPA and DI‐GM levels, the prevalence of hypertension was highest in the lower‐level LTPA (< 150 min/week) and lower DI‐GM (≤ 4 points) group and significantly decreased in the higher‐level LTPA (≥ 150 min/week) and higher DI‐GM (> 4 points) group. Similarly, among different groups of participants with various LTPA patterns and DI‐GM combinations, the prevalence of hypertension was highest in the inactive population with DI‐GM ≤ 4, and significantly decreased in the regularly active population with DI‐GM > 4 (Figure 2C,D).

**TABLE 1:** The baseline characteristics of participants.

Variables	Overall (*n* = 27,643)	DI‐GM scores				
		0–3 (*n* = 6785)	4 (*n* = 6806)	5 (*n* = 6377)	≥ 6 (*n* = 7675)	*p*
**Age **(**years**)	47.29 ± 0.26	45.25 ± 0.35	45.82 ± 0.33	47.56 ± 0.36	49.74 ± 0.36	< 0.0001
**Sex, *n* (%)**						< 0.0001
Female	13,835 (50.64)	3190 (21.07)	3348 (22.47)	3207 (23.95)	4090 (32.50)	
Male	13,808 (49.36)	3595 (24.30)	3458 (24.35)	3170 (23.24)	3585 (28.11)	
**Race, *n* (%)**						< 0.0001
Non‐Hispanic White	11,973 (68.25)	2691 (21.12)	2759 (22.40)	2764 (23.88)	3759 (32.59)	
Non‐Hispanic Black	5758 (10.17)	1814 (32.31)	1508 (25.64)	1268 (21.97)	1168 (20.07)	
Mexican Americans	3961 (8.28)	912 (23.82)	1104 (27.81)	991 (26.00)	954 (22.37)	
Other	5951 (13.31)	1368 (22.52)	1435 (24.06)	1354 (21.91)	1794 (31.51)	
**Education level, *n* (%)**						< 0.0001
High school or more	21,793 (86.58)	5176 (21.84)	5124 (22.61)	5077 (23.85)	6416 (31.70)	
Less than high school	5850 (13.42)	1609 (28.00)	1682 (28.52)	1300 (22.02)	1259 (21.47)	
**Marital status, *n* (%)**						< 0.0001
Married or living with a partner	16,499 (62.63)	3883 (21.17)	3924 (22.90)	3893 (24.04)	4799 (31.89)	
Not married nor living with a partner	11,144 (37.37)	2902 (25.18)	2882 (24.23)	2484 (22.87)	2876 (27.71)	
**FPIR, *n* (%)**						< 0.0001
< 1.3	8350 (21.03)	2338 (27.90)	2312 (26.60)	1886 (23.31)	1814 (22.19)	
1.3–3.5	10,402 (34.35)	2745 (24.78)	2567 (24.36)	2340 (22.27)	2750 (28.59)	
≥ 3.5	8891 (44.62)	1702 (18.58)	1927 (21.15)	2151 (24.76)	3111 (35.50)	
**Smoke Status, *n* (%)**						< 0.0001
Never/Former	22,065 (80.63)	5174 (21.84)	5314 (22.69)	5093 (23.65)	6484 (31.82)	
Now	5578 (19.37)	1611 (26.10)	1492 (26.35)	1284 (23.42)	1191 (24.14)	
**Alcohol Use** **,** ***n*** **(%)**						0.33
Never/Former	7141 (20.68)	1730 (23.06)	1802 (24.24)	1650 (23.70)	1959 (29.01)	
Current	20,502 (79.32)	5055 (22.57)	5004 (23.18)	4727 (23.58)	5716 (30.67)	
**BMI (kg/m^2^)**						< 0.0001
< 30	16,641 (61.46)	3763 (20.63)	4046 (22.45)	3833 (23.88)	4999 (33.03)	
≥ 30	11,002 (38.54)	3022 (25.92)	2760 (24.91)	2544 (23.16)	2676 (26.02)	
**CVD, *n* (%)**						0.23
No	24,699 (91.49)	6028 (22.50)	6082 (23.53)	5704 (23.62)	6885 (30.35)	
Yes	2944 (8.51)	757 (24.50)	724 (21.99)	673 (23.42)	790 (30.08)	
**Diabetes, *n* (%)**						< 0.0001
No	22,460 (85.91)	5376 (22.05)	5507 (23.44)	5235 (23.72)	6342 (30.79)	
Yes	5183 (14.09)	1409 (26.42)	1299 (23.15)	1142 (22.89)	1333 (27.53)	
**Cancer, *n* (%)**						< 0.0001
No	24,991 (89.84)	6204 (23.02)	6228 (23.74)	5738 (23.41)	6821 (29.82)	
Yes	2652 (10.16)	581 (19.53)	578 (20.39)	639 (25.27)	854 (34.81)	
**eGFR** (**mL** **/** **min**/**1.73** **m** ^**2**^)	94.38 ± 0.32	96.15 ± 0.42	95.70 ± 0.42	93.83 ± 0.50	92.48 ± 0.43	< 0.0001
**Energy** (**Kcal**)	2163.86 ± 8.22	2187.27 ± 17.53	2116.91 ± 17.11	2137.91 ± 16.97	2202.77 ± 15.47	< 0.001
**BGMS**	2.15 ± 0.02	0.95 ± 0.02	1.53 ± 0.02	2.21 ± 0.02	3.47 ± 0.02	< 0.0001
**UGBS**	2.57 ± 0.01	1.59 ± 0.02	2.47 ± 0.02	2.79 ± 0.02	3.19 ± 0.01	< 0.0001
**Hypertension, *n* (%)**						0.23
No	12,590 (50.00)	2976 (21.95)	3149 (23.64)	2923 (23.64)	3542 (30.77)	
Yes	15,053 (50.00)	3809 (23.38)	3657 (23.16)	3454 (23.56)	4133 (29.89)	
**Total PA** (**min/week**)	1006.20 ± 17.34	1112.27 ± 31.32	1068.83 ± 30.74	1005.62 ± 29.23	879.07 ± 22.25	< 0.0001
**LTPA (min/week), *n* (%)**						< 0.0001
< 150	18,274 (60.78)	4775 (24.91)	4702 (24.84)	4169 (23.14)	4628 (27.10)	
≥ 150	9369 (39.22)	2010 (19.19)	2104 (21.16)	2208 (24.32)	3047 (35.33)	
**LTPA (min/week), *n* (%)**						< 0.0001
0	14,117 (44.44)	3839 (26.09)	3735 (25.39)	3190 (23.25)	3353 (25.27)	
1–149	4157 (16.34)	936 (21.71)	967 (23.37)	979 (22.85)	1275 (32.07)	
150–299	2928 (11.77)	629 (20.96)	639 (18.72)	722 (25.84)	938 (34.48)	
≥ 300	6441 (27.45)	1381 (18.43)	1465 (22.21)	1486 (23.66)	2109 (35.70)	
**LTPA pattern, *n* (%)**						< 0.0001
Inactive	14,117 (44.44)	3839 (26.09)	3735 (25.39)	3190 (23.25)	3353 (25.27)	
Insufficiently active	4157 (16.34)	936 (21.71)	967 (23.37)	979 (22.85)	1275 (32.07)	
Weekend warrior	1162 (4.47)	280 (24.04)	302 (23.41)	322 (29.43)	258 (23.11)	
Regularly active	8207 (34.76)	1730 (18.56)	1802 (20.87)	1886 (23.66)	2789 (36.91)	

*Note:* Continuous variables were presented as mean ± SE, and categorical variables were presented as the frequency with percentage.

Abbreviations: BGMS, beneficial to gut microbiota score; BMI, body mass index; CI, confidence interval; CVD, cardiovascular disease; DI‐GM, dietary index for gut microbiota; eGFR, estimated glomerular filtration rate; FPIR, family poverty income ratio; OR, odds ratio; UGMS, unfavorable to gut microbiota score.

### 3.2 Association Between DI‐GM, LTPA and Hypertension Among the NHANES 2007–2020 Participants

**FIGURE 3:**
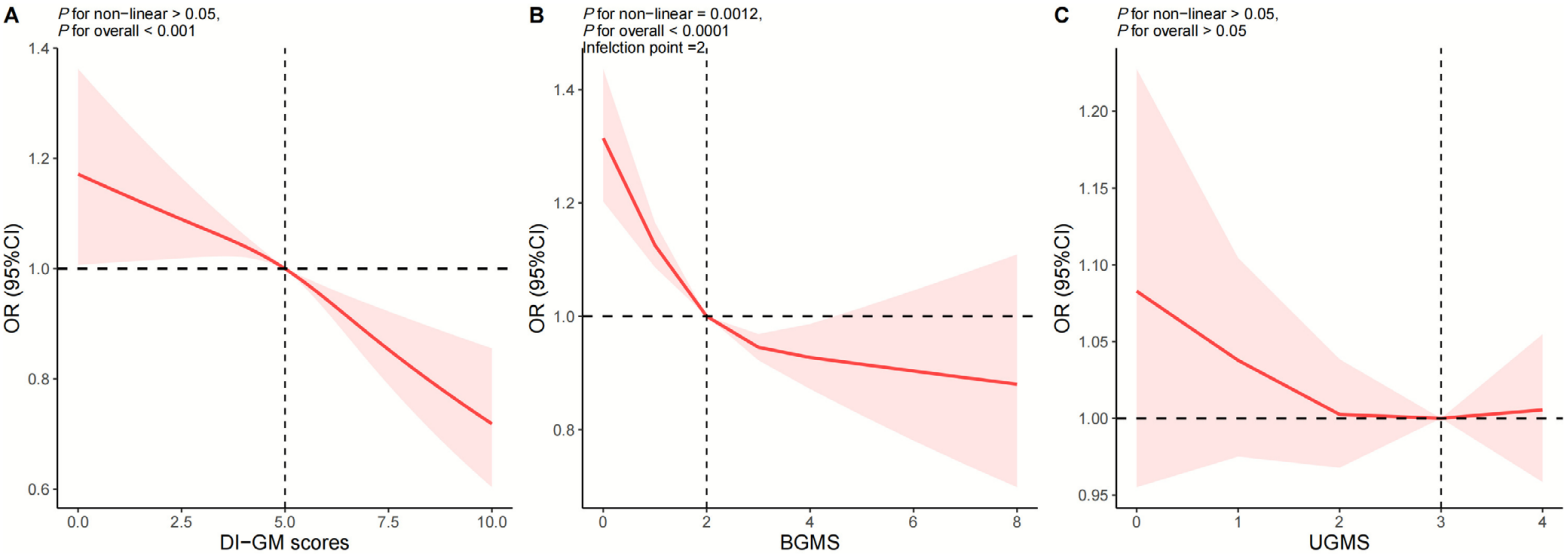
Restricted cubic spline analysis (RCS) with multivariate‐adjusted associations between DI‐GM and the risk of hypertension. (A) RCS analysis between DI‐GM and hypertension. (B) RCS analysis between BGMS and hypertension. (C) RCS analysis between UGMS and hypertension. All RCS analysis adjusted for age, sex, and race/ethnicity, education level, marital status, FPIR, smoking status, alcohol drinking status, total physical activity, CVD, diabetes, eGFR, BMI, and energy intake. BGMS, beneficial to gut microbiota score; BMI, body mass index; CI, confidence interval; CVD, cardiovascular disease; DI‐GM, dietary index for gut microbiota. DI‐GM, dietary index for gut microbiota; eGFR, estimated glomerular filtration rate; FPIR, family poverty income ratio; LTPA, leisure time physical activity; OR, odds ratio; UGMS, unfavorable to gut microbiota score.

As shown in Table 2, both univariate and multivariate logistic regression analyses demonstrated consistent and stable associations between DI‐GM and hypertension across unadjusted, partially adjusted, and fully adjusted models. Results revealed a significant inverse correlation between DI‐GM scores and hypertension risk. To validate the robustness of these results, DI‐GM was analyzed as a categorical variable in logistic regression. In Model 2, the highest DI‐GM group was associated with progressively lower hypertension risk, compared with the lowest DI‐GM group. Specifically, participants with DI‐GM scores ≥ 6 demonstrated a 16% lower risk of hypertension compared to those scoring 0–3 (OR = 0.84, 95% CI: 0.76–0.94), with the difference reaching statistical significance. Subsequent analyses evaluated the components of DI‐GM (BGMS and UGMS) in relation to hypertension. Multivariate logistic regression indicated a significant inverse association between BGMS and hypertension, whereas UGMS showed no significant relationship (Table 2). Furthermore, multivariable‐adjusted RCS analysis showed a linear dose–response relationship between DI‐GM and hypertension risk (*p* for non‐linear > 0.05, Figure 3A). Notably, BGMS exhibited a nonlinear association with hypertension (*p* for non‐linear = 0.0012, Figure 3B), while no significant correlation was observed between UGMS and hypertension (Figure 3C).

**TABLE 2:** Association between DI‐GM and hypertension among the NHANES 2007–2020 participants.

Characteristics	Crude model		Model 1		Model 2	
	OR (95% CI)	*p*	OR (95% CI)	*p*	OR (95% CI)	*p*
**DI‐GM scores**	0.98 (0.96, 1.00)	0.02	0.92 (0.90, 0.94)	< 0.0001	0.95 (0.93, 0.98)	< 0.001
**DI‐GM group**						
0–3	Reference		Reference		Reference	
4	0.92 (0.83, 1.02)	0.11	0.90 (0.80, 1.00)	0.05	0.94 (0.84, 1.06)	0.31
5	0.94 (0.85, 1.03)	0.18	0.84 (0.75, 0.94)	0.002	0.92 (0.82, 1.03)	0.15
≥ 6	0.91 (0.84, 0.99)	0.03	0.73 (0.66, 0.80)	< 0.0001	0.84 (0.76, 0.94)	0.003
***p* for trend**		0.34		0.39		0.56
BGMS	0.97 (0.95, 1.00)	0.05	0.92 (0.90, 0.95)	< 0.0001	0.93 (0.90, 0.96)	< 0.0001
UGBS	0.98 (0.95, 1.01)	0.29	0.94 (0.90, 0.97)	0.001	0.99 (0.95, 1.03)	0.58

*Note:* The crude model was not adjusted for any covariates. Model 1 = age, sex, and race/ethnicity. Model 2 = Model 1 + (education level, marital status, FPIR, smoking status, alcohol drinking status, total physical activity, CVD, diabetes, eGFR, BMI, and energy intake). Abbreviations: BGMS, beneficial to gut microbiota score; BMI, body mass index; CI, confidence interval; CVD, cardiovascular disease; DI‐GM, dietary index for gut microbiota; eGFR, estimated glomerular filtration rate; FPIR, family poverty income ratio; OR, odds ratio; UGMS, unfavorable to gut microbiota score.

Similarly, we further examined the relationship between LTPA and hypertension across different durations and patterns. Our findings revealed a significant inverse association between LTPA and hypertension in fully adjusted logistic regression models. To explore the dose–response relationship, we categorized LTPA into four groups: 0 min/week, 1–149 min/week, 150–299 min/week, and ≥ 300 min/week. Compared to individuals with no LTPA, those engaging in ≥ 150 min/week of LTPA showed significantly reduced hypertension incidence. Specifically, the 150–299 min/week group demonstrated a 17% lower hypertension risk (OR = 0.83, 95% CI: 0.72–0.96), while the ≥ 300 min/week group exhibited a 26% risk reduction (OR = 0.74, 95% CI: 0.65–0.83) (Table 3). Multivariable‐adjusted RCS analysis showed a non‐linear dose–response relationship between LTPA and hypertension risk (*p* for non‐linear < 0.001, Figure S2).

**TABLE 3:** Association between LTPA intensity, pattern and hypertension.

Characteristics	Crude model		Model 1		Model 2	
	OR (95% CI)	*p*	OR (95% CI)	*p*	OR (95% CI)	*p*
**LTPA** (**min** **/** **week**) **≥** **150** **vs** **< ** **150**	0.54 (0.50, 0.59)	< 0.0001	0.68 (0.62, 0.74)	< 0.0001	0.79 (0.72, 0.87)	< 0.0001
**LTPA (min/week)**						
0	Reference		Reference		Reference	
1–149	0.73 (0.65, 0.82)	< 0.0001	0.83 (0.73, 0.93)	0.003	0.90 (0.79, 1.02)	0.10
150–299	0.62 (0.55, 0.71)	< 0.0001	0.72 (0.62, 0.83)	< 0.0001	0.83 (0.72, 0.96)	0.01
≥ 300	0.45 (0.41, 0.49)	< 0.0001	0.62 (0.55, 0.68)	< 0.0001	0.74 (0.65, 0.83)	< 0.0001
*p* for trend		< 0.0001		< 0.0001		< 0.0001
**LTPA pattern**						
Inactive	Reference		Reference		Reference	
Insufficiently active	0.73 (0.65, 0.82)	< 0.0001	0.83 (0.73, 0.93)	0.003	0.90 (0.79, 1.02)	0.10
Weekend warrior	0.56 (0.47, 0.66)	< 0.0001	0.70 (0.57, 0.86)	< 0.001	0.80 (0.64, 0.99)	0.04
Regularly active	0.49 (0.45, 0.53)	< 0.0001	0.64 (0.58, 0.70)	< 0.0001	0.76 (0.69, 0.84)	< 0.0001

*Note:* The crude model was not adjusted for any covariates. Model 1 = age, sex, and race/ethnicity. Model 2 = Model 1 + (education level, marital status, FPIR, smoking status, alcohol drinking status, total physical activity, CVD, diabetes, eGFR, BMI, and energy intake). Abbreviations: BMI, body mass index; CI, confidence interval; CVD, cardiovascular disease; DI‐GM, dietary index for gut microbiota; eGFR, estimated glomerular filtration rate; FPIR, family poverty income ratio; LTPA, leisure time physical activity; OR, odds ratio.

Additionally, we evaluated the relationship between different LTPA patterns (based on exercise intensity and weekly frequency) and hypertension. Both the weekend warrior pattern (OR = 0.80, 95% CI: 0.64–0.99, *p* = 0.04) and regularly active pattern (OR = 0.76, 95% CI: 0.69–0.84, *p* < 0.0001) maintained significant protective associations after full adjustment. However, the association for insufficiently active individuals was attenuated and non‐significant in Model 2 (OR = 0.90, 95% CI: 0.79–1.02, *p* = 0.10). These findings underscore the importance of both achieving sufficient activity volume and distributing exercise sessions regularly throughout the week for optimal hypertension prevention.

Next, we examined the relationship between DI‐GM and hypertension among individuals meeting or not meeting the PA guideline recommendations. In the logistic regression model adjusted for all covariates, we found that among those with insufficient LTPA (not meeting PA guidelines), individuals with DI‐GM ≥ 6 exhibited a 19% significantly lower risk of hypertension compared to the lowest DI‐GM group (OR = 0.81, 95% CI: 0.71–0.92). In contrast, among individuals meeting the PA guidelines, DI‐GM showed no association with hypertension risk, with all *p*‐values ≥ 0.05 (Table 4).

**TABLE 4:** Association between DI‐GM and hypertension among participants with different intensities of LTPA.

Characteristics	Crude model		Model 1		Model 2	
	OR (95% CI)	*p*	OR (95% CI)	*p*	*p*	*p*
**LTPA (min/week) < 150** **DI‐GM group**						
0–3	Reference		Reference		Reference	
4	0.89 (0.79, 0.99)	0.04	0.85 (0.75, 0.96)	0.01	0.89 (0.78, 1.01)	0.07
5	0.93 (0.82, 1.06)	0.28	0.82 (0.71, 0.95)	0.01	0.88 (0.76, 1.02)	0.08
≥ 6	0.98 (0.89, 1.09)	0.74	0.73 (0.65, 0.83)	< 0.0001	0.81 (0.71, 0.92)	0.002
*p* for trend		0.37		0.27		0.4
**LTPA (min/week) ≥ 150** **DI‐GM group**						
0–3	Reference		Reference		Reference	
4	1.02 (0.84, 1.25)	0.83	1.03 (0.84, 1.26)	0.78	1.06 (0.86, 1.31)	0.59
5	1.06 (0.90, 1.26)	0.49	0.96 (0.80, 1.15)	0.64	1.04 (0.85, 1.26)	0.71
≥ 6	1.00 (0.86, 1.17)	0.97	0.85 (0.71, 1.00)	0.05	0.98 (0.82, 1.18)	0.85
*p* for trend		0.54		0.16		0.67

*Note:* The crude model was not adjusted for any covariates. Model 1 = age, sex, and race/ethnicity. Model 2 = Model 1 + (education level, marital status, FPIR, smoking status, alcohol drinking status, total physical activity, CVD, diabetes, eGFR, BMI, and energy intake). Abbreviations: BMI, body mass index; CI, confidence interval; CVD, cardiovascular disease; DI‐GM, dietary index for gut microbiota; eGFR, estimated glomerular filtration rate; FPIR, family poverty income ratio; LTPA, leisure time physical activity; OR, odds ratio.

### 3.3 Joint Analysis of DI‐GM and LTPA With Hypertension

**FIGURE 4:**
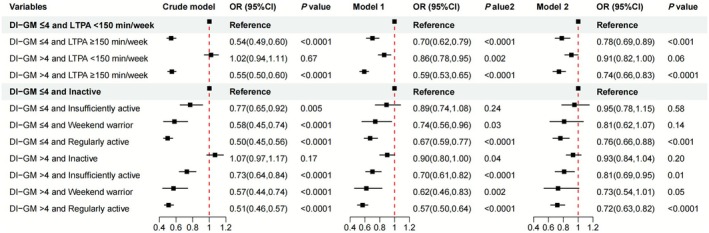
Joint effects of DI‐GM and LTPA on hypertension. The crude model was not adjusted for any covariates. Model 1 = age, sex, and race/ethnicity. Model 2 = Model 1 + (education level, marital status, FPIR, smoking status, alcohol drinking status, total physical activity, CVD, diabetes, eGFR, BMI, and energy intake). BMI, body mass index; CI, confidence interval; CVD, cardiovascular disease; DI‐GM, dietary index for gut microbiota; eGFR, estimated glomerular filtration rate; FPIR, family poverty income ratio; LTPA, leisure time physical activity; OR, odds ratio.

Finally, we analyzed the combined effects of DI‐GM and LTPA duration/patterns on hypertension. In a fully adjusted multivariable regression model, individuals with LTPA ≥ 150 min/week and DI‐GM > 4 demonstrated a 26% lower hypertension risk (OR = 0.74, 95% CI: 0.66–0.83) compared to those with LTPA < 150 min/week and DI‐GM ≤ 4 (Figure 4). Subsequent risk reductions were observed in: (1) LTPA ≥ 150 min/week and DI‐GM ≤ 4: 22% lower risk (OR = 0.78, 95% CI: 0.69–0.89); (2) LTPA < 150 min/week and DI‐GM > 4: 9% lower risk (OR = 0.91, 95% CI: 0.82–1.00) (Figure 4).

Analysis of LTPA patterns combined with DI‐GM demonstrated that regularly active individuals with DI‐GM > 4 had the most pronounced reduction in hypertension risk—28% lower (OR = 0.72, 95% CI: 0.63–0.82)—compared to inactive individuals with DI‐GM ≤ 4. Critically, regardless of LTPA duration or pattern, individuals with DI‐GM > 4 consistently showed a lower hypertension risk than those with DI‐GM ≤ 4, underscoring the independent and additive protective role of higher DI‐GM levels (Figure 4).

### 3.4 Subgroup Analyses

**FIGURE 5:**
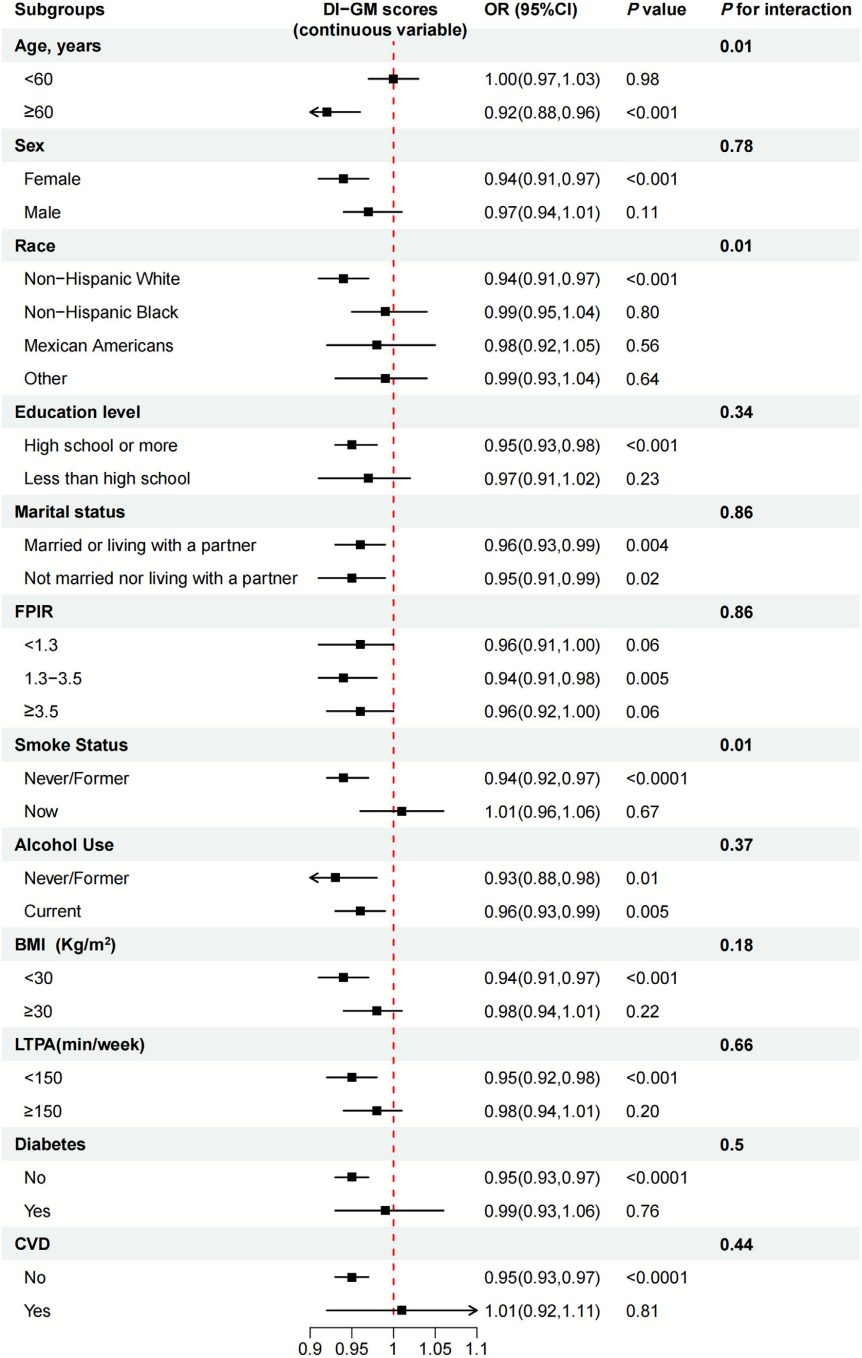
Subgroup analyses of the association between DI‐GM and hypertension. The analyses were adjusted for age, sex, race/ethnicity, education level, marital status, FPIR, smoking status, alcohol drinking status, total physical activity, CVD, diabetes, eGFR, BMI, and energy intake. When stratified separately, stratified variables are not included. BMI, body mass index; CI, confidence interval; CVD, cardiovascular disease; DI‐GM, dietary index for gut microbiota; eGFR, estimated glomerular filtration rate; FPIR, family poverty income ratio; OR, odds ratio.

Figure 5 showed that significant protective effects of DI‐GM (continuous variables) were observed across most subgroups, particularly in individuals aged ≥ 60 years, females, Non‐Hispanic Whites, high school or more education level, married or living with a partner, FPIR within 1.3‐3.5, nerver/former smoking, and those with BMI < 30, and without diabetes or CVD. Interaction tests revealed significantly modified effects by smoking status (*p* for interaction = 0.01; no benefit in current smokers) and race (*p* for interaction = 0.01; no significant benefit in Non‐Hispanic Blacks, Mexican Americans subgroups, or others). Additionally, after multiple imputation, the association between DI‐GM and hypertension remained significant (Table S1). Figure S3 indicated that LTPA (≥ 150 vs. < 150 min/week) exerted significant protective effects in most subgroups.

## 4 Discussion

This study investigated the independent and joint effects of diet‐gut microbiota function (assessed through the Dietary Index‐Gut Microbiota, DI‐GM) and LTPA on hypertension risk using a nationally representative U.S. database. Results demonstrated that both higher DI‐GM scores and elevated LTPA levels were significantly associated with reduced hypertension prevalence. Combined effect analysis revealed the lowest risk of hypertension among individuals with optimal DI‐GM scores (≥ 6 points) and higher LTPA engagement (> 150 min/week). Notably, populations with insufficient PA exhibited enhanced protective benefits from improved DI‐GM scores, suggesting a potential interactive mechanism through which dietary‐microbiota interactions and physical activity collectively influence blood pressure regulation.

### 4.1 DI‐GM Exhibits an Independent Association With Hypertension

The protective relationship between the DI‐GM index and hypertension may stem from dietary regulation of gut microbiota function. Emerging research highlights the critical role of this cross‐system interaction in the pathophysiology of hypertension (Chen et al. 2023; Yang et al. 2023; Sircana et al. 2019). Key mechanisms involve gut microbiota‐derived metabolites, such as short‐chain fatty acids, which influence host metabolism, energy absorption, intestinal barrier integrity, systemic inflammation, vascular endothelial function, and activity of the renin‐angiotensin‐aldosterone system (RAAS) (O'Donnell et al. 2023; Gao et al. 2024; Li et al. 2017). Targeting the gut microbiota represents a novel therapeutic approach for hypertension prevention and management. Current strategies include dietary modifications, probiotics, fecal microbiota transplantation, and antibiotics (Avery et al. 2021; Mahgoup 2025) with dietary intervention being the most direct and practical approach (Jama et al. 2019). Evidence and clinical guidelines underscored that hypertension‐preventive diets, such as the Mediterranean diet and the Dietary Approaches to Stop Hypertension (DASH), significantly reduce hypertension risk by providing nutrients such as calcium, fiber, and antioxidants (Filippou et al. 2023; Pagidipati et al. 2024). These diets focus on vegetables, fruits, and whole grains, low sodium, minimal processed foods, and lean protein sources, instead of red meat, creating a synergistic dietary framework for reducing hypertension (Craddick et al. 2003; Davis et al. 2015).

The DI‐GM index focused on fermented foods, specific functional components like cranberries, coffee, and green tea, and integrated core elements of the DASH and Mediterranean diets, such as plant‐based meals, healthy fats, and high fiber intake (Kase et al. 2024). These dietary components regulate gut microbiota to manage hypertension. For instance, whole grains and fiber increase gut microbiota diversity, stimulating the production of SCFAs like propionate and butyrate (van der Hee and Wells 2021). These metabolites activate G protein‐coupled receptors (GPR41/43), suppress renin‐angiotensin system (RAS) activity, improve vascular smooth muscle elasticity, and reduce vasoconstriction (Samuel et al. 2008; Maslowski et al. 2009). A key feature of DI‐GM is its inclusion of fermented dairy products, which provide probiotics that lower blood pressure. During fermentation, probiotics generate bioactive peptides, such as ACE‐inhibitory peptides, that exhibit antioxidant, antibacterial, and antihypertensive effects via ACE enzyme inhibition (Martin et al. 2015; Rendón‐Rosales et al. 2022).

It should be emphasized that the protective association between DI‐GM and hypertension is likely mediated through multiple biological pathways. Although the present study focuses on gut microbiota‐related mechanisms, it is noteworthy that many components of the DI‐GM—such as fruits, vegetables, whole grains, green tea, and fermented foods—are established sources of cardioprotective nutrients (e.g., polyphenols, antioxidants, potassium, and dietary fiber) (Jaffe 2013). These compounds may confer benefits through direct physiological effects, including enhanced endothelial function, reduced oxidative stress, and improved vasodilation, independent of microbial modulation. Thus, DI‐GM reflects a composite dietary pattern that engages both microbiome‐dependent and conventional cardiometabolic pathways. Future studies integrating metagenomics, targeted metabolomics, and mediation analysis are warranted to quantify the relative contribution of gut microbiota to these benefits and elucidate potential synergies among mechanisms.

It is noteworthy that, in contrast to the clearly beneficial associations observed for the overall DI‐GM and the BGMS, our analysis did not identify a significant independent relationship between the UGMS and hypertension risk. This lack of association may be attributed to the narrower dietary scope and reverse ‐scoring methodology of the UGMS; more broadly, it suggests that the overall quality and balance of the diet, as captured by the composite DI‐GM, may matter more for blood pressure than the isolated avoidance of detrimental foods.

We also identified a significant racial disparity in the association between DI‐GM and hypertension (*p* for interaction = 0.01), with protective effects evident only among non‐Hispanic White individuals and absent in other race participants. This heterogeneity may be attributed to several interrelated factors. First, well‐established differences in genetic susceptibility to hypertension and salt sensitivity—more prevalent among Black populations (Svetkey et al. 1996; Aburto et al. 2013; Manosroi et al. 2017)—may dominate cardiovascular risk pathways, thereby attenuating the modulatory influence of diet and microbiome‐mediated mechanisms. Second, the DI‐GM data were developed based on general U.S. dietary habits and may lack transcultural applicability, as it does not fully capture culturally specific foods, dietary patterns, or traditional cooking methods that influence both dietary quality and microbial composition. Third, variations in gut microbiota structure across ethnic groups, shaped by long‐term dietary traditions, host genetics, and socioeconomic factors—such as access to healthy foods and educational attainment—may contribute to differential responses to dietary interventions (Parizadeh and Arrieta 2023). Furthermore, the greater burden of social determinants of health in minority populations—including structural inequities and disparities in healthcare access—directly exacerbates hypertension risk and promotes unhealthy behavioral patterns (e.g., higher smoking rates) (Carey et al. 2022). These multifaceted disadvantages may collectively offset the dietary benefits captured by the DI‐GM. Therefore, future studies should prioritize the development of culturally adapted dietary indices and support longitudinal interventions in diverse racial and ethnic cohorts to elucidate the ethnic heterogeneity underlying the diet–microbiota–blood pressure axis and advance equitable, precision nutrition strategies for hypertension prevention.

### 4.2 Hypotensive Effects and Variability of LTPA

LTPA has been consistently associated with hypertension prevention and better cardiovascular health in individuals with high blood pressure (Souza et al. 2024; Cho et al. 2024; Maruf and Ucheokoye 2023; Al Kibria et al. 2022). Our findings also found that participants engaging in ≥ 300 min/week of LTPA had a 26% lower hypertension risk compared to those not meeting PA guidelines (OR = 0.74, 95% CI: 0.65%–0.83%), with a significant dose–response trend (*p* for trend < 0.001).

However, as observed by (Tu et al. 2024), the benefits of LTPA depend on context. For instance, individuals following an anti‐inflammatory diet showed lower hypertension prevalence and reduced cardiovascular mortality risk compared to those on pro‐inflammatory diets, even with low physical activity levels. Interestingly, our study found that after the time of LTPA reached PA guideline, there was no longer a statistical difference between DI‐GM and the risk of hypertension among the participants. In contrast, the high DI‐GM group demonstrated a more obvious reduction in hypertension risk among those with inadequate LTPA, suggesting that optimizing dietary patterns to enhance gut microbiota function may offset blood pressure dysregulation caused by insufficient physical activity. This heterogeneity may be related to the characteristics of physical activity itself. Among people who are not meeting PA guidelines, they may rely more on the protective effects brought by diet and gut microbiota. On the contrary, among people who have met PA guidelines, PA itself directly or indirectly reduces the risk of hypertension by improving vascular endothelial function, promoting vasodilation, and improving insulin sensitivity and metabolic syndrome, etc. Furthermore, regular exercise promotes the growth of beneficial bacteria (e.g., *Akkermansia muciniphila, AKK*) (Fontana et al. 2023; Clarke et al. 2014), which synergistically enhance metabolic benefits, creating a positive feedback loop (Munukka et al. 2018). These effects may offset the protective effect of DI‐GM. These findings highlight DI‐GM's critical role in blood pressure control, especially for individuals unable to meet PA guidelines.

At present, many literatures have proved that the results are convincing, emphasizing that diet‐gut microbiota function regulation and sufficient PA are associated with a reduced prevalence of hypertension (Xia et al. 2021; Marques et al. 2017; Zhu and Wang 2024; Liu et al. 2017). However, our study, the first to investigate the combined influence of diet, gut microbiota, and LTPA on hypertension, demonstrated that individuals with both higher DI‐GM scores (> 4 points) and sufficient LTPA (≥ 150 min/week) had a 26% lower hypertension risk compared to the reference group (OR = 0.74, 95% CI: 0.66–0.83). Further analysis revealed that participants with regular LTPA pattern habits and higher DI‐GM scores (> 4 points) showed the strongest protective effect (OR = 0.72, 95% CI: 0.63–0.82), underscoring a significant joint effect between diet‐driven microbiota modulation and regular PA.

Our study was the first to identify the potential synergistic effects between PA and diet–microbiota interactions in both preventing and managing hypertension. It presented a novel approach to hypertension prevention by demonstrating that dietary interventions aimed at optimizing gut microbiota function may compensate for insufficient LTPA, particularly in individuals falling short of recommended activity levels. These findings support the implementation of an integrated “diet‐exercise‐microbiota” strategy to improve blood pressure regulation, providing a comprehensive framework for cardiovascular health interventions.

## 5 Clinical Implications and Limitations

This study is the first to confirm the combined role of DI‐GM and LTPA in reducing hypertension risk, offering novel insights for personalized prevention strategies: optimizing dietary patterns and promoting regular LTPA may enhance blood pressure control. For individuals with limited LTPA capacity (e.g., older adults or those with chronic conditions), optimizing dietary patterns to enhance gut microbiota function may serve as an effective alternative strategy, while prioritizing dietary quality remains critical for highly active populations to maximize health benefits.

This study has several limitations that warrant consideration. First, owing to its cross‐sectional design using NHANES data, the observed associations between DI‐GM, LTPA, and hypertension are indicative of correlation rather than causation. Moreover, reverse causality remains a concern; for example, individuals with a hypertension diagnosis may have adopted healthier dietary and physical activity patterns following clinical advice, which could subsequently affect both DI‐GM scores and LTPA behavior. Thus, the observed associations may partly reflect post‐diagnosis adaptations rather than pre‐disease risk profiles. Longitudinal or interventional studies are needed to establish temporal order and causal inference. Second, although we adjusted for numerous potential confounders, residual confounding may persist due to unmeasured variables such as genetic susceptibility, psychosocial stress, or detailed medication history, which could influence both exposure and outcome.

Third, physical activity assessment was limited to leisure‐time activities and did not account for other domains (e.g., occupational, transport‐related, or household activities). This may lead to incomplete quantification of total physical activity and potential underestimation of its association with hypertension. Future research should incorporate objective measures (e.g., accelerometry) and multi‐domain physical activity assessment to better capture overall activity exposure. Fourth, 8865 participants were excluded due to missing covariate data, which could introduce selection bias. Direct comparison of baseline characteristics between included and excluded participants was not feasible, as the latter lacked essential baseline data. However, sensitivity analysis (Figure 5; Figure S3; Table S1) confirmed that our primary findings remained robust to the exclusion of these participants, alleviating concerns about significant bias. Finally, the DI‐GM index was derived from selected dietary and microbial databases, and its generalizability across diverse populations remains to be established. External validation in varied demographic and cultural settings is necessary to confirm its broader applicability.

## 6 Conclusion

In this population‐based cohort study, both a high DI‐GM score (≥ 6 points) and sufficient LTPA (≥ 150 min/week) independently reduced hypertension risk, with their combined effect amplifying protective benefits. Notably, a high DI‐GM score significantly lowered hypertension risk even in individuals with insufficient LTPA, highlighting the compensatory potential of diet‐microbiota regulation. By jointly evaluating these factors, our findings unveil a synergistic “diet‐exercise‐microbiota” mechanism and propose a novel strategy for hypertension prevention.

## Author Contributions

J.W., X.L., Y.Z. and Z.J. contributed to the conception and research design. J.W. and X.L. performed the analysis and interpretation of data. X.Z., Z.W. and J.W. contributed to the writing, review, and revision of the manuscript. All authors participated in the design of the research and the review of the manuscript. X.Z. and Z.W. contributed to the study supervision. The authors read and approved the final manuscript.

## Funding

This study was funded by Guangdong Provincial Administration of Traditional Chinese Medicine Research Project (20251084), Guangdong Basic and Applied Basic Research Foundation (2023A1515011896), and Young Talent Support Project of Guangzhou Association for Science and Technology (QT‐2025‐040).

## Ethics Statement

The NHANES study protocol was approved by the NCHS Research Ethics Review Board.

## Consent

Written informed consent was obtained from all study participants.

## Conflicts of Interest

The authors declare no conflicts of interest.

## Supporting information


**Figure S1** Standardized variance inflation factor across key study variables.

**Figure S2:** Restricted cubic spline analysi with multivariate‐adjusted associations between LTPA and the risk of hypertension.

**Figure S3:** Subgroup analyses of the association between LTPA and hypertension.

**Table S1:** Association between DI‐GM and hypertension among the NHANES 2007–2020 participants after multiple imputation.

## Data Availability

The data that support the findings of this study are available in NHANES at https://www.cdc.gov/nchs/nhanes/index.htm.
