# Supplementary figures and images for: Dietary Index for Gut Microbiota and Leisure Time Physical Activity: The Potential Combined Protective Impact on Hypertension Risk
Source: Food Sci Nutr. 2025 Nov 27;13(12):e71245. doi: 10.1002/fsn3.71245 (PMC12661085; doi:10.1002/fsn3.71245)

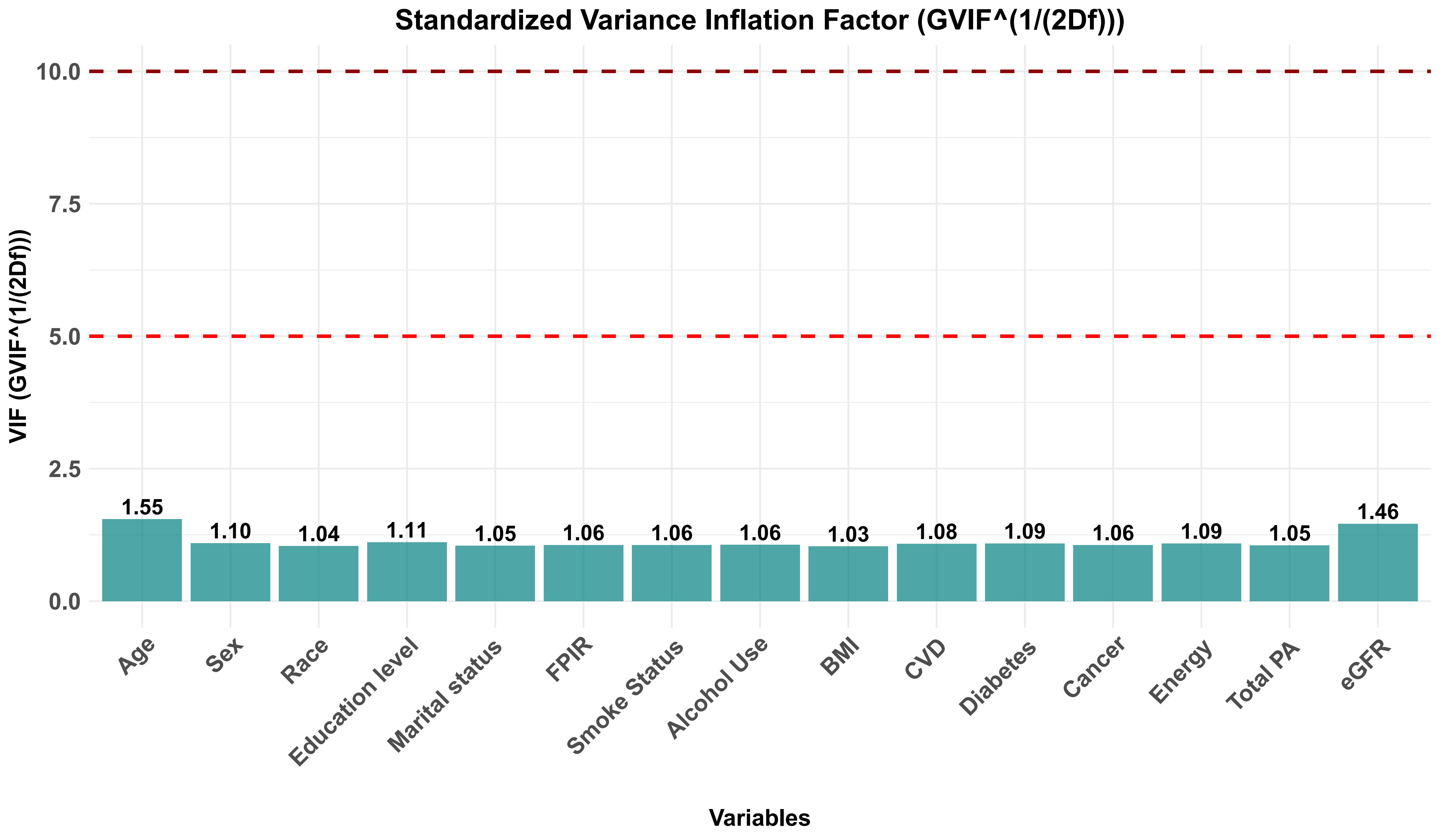

Supplement: Supplementary file 1 — Figure S1 Standardized variance inflation factor across key study variables. [file FSN3-13-e71245-s002.tif]

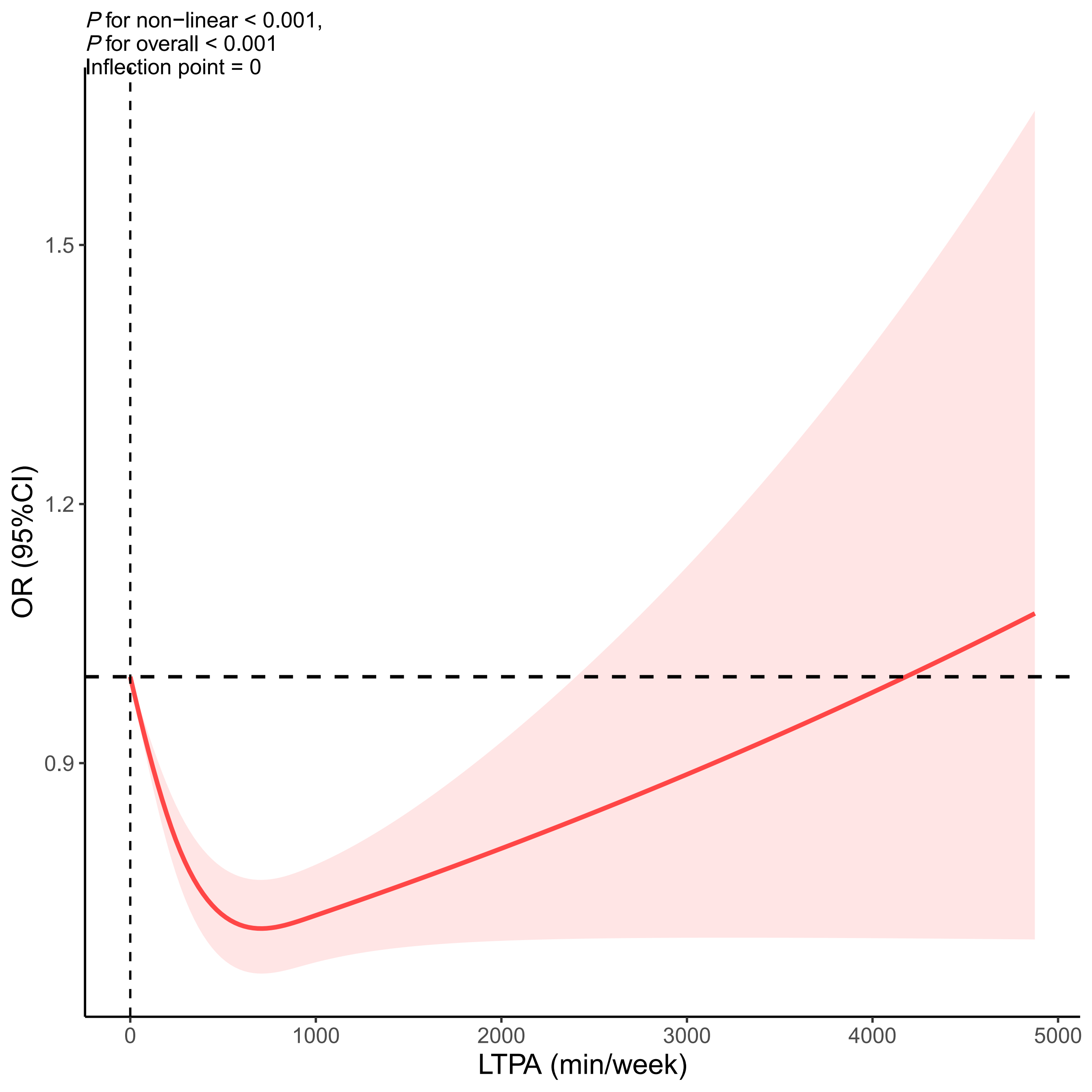

Supplement: Supplementary file 2 — Figure S2: Restricted cubic spline analysi with multivariate‐adjusted associations between LTPA and the risk of hypertension. [file FSN3-13-e71245-s004.tif]

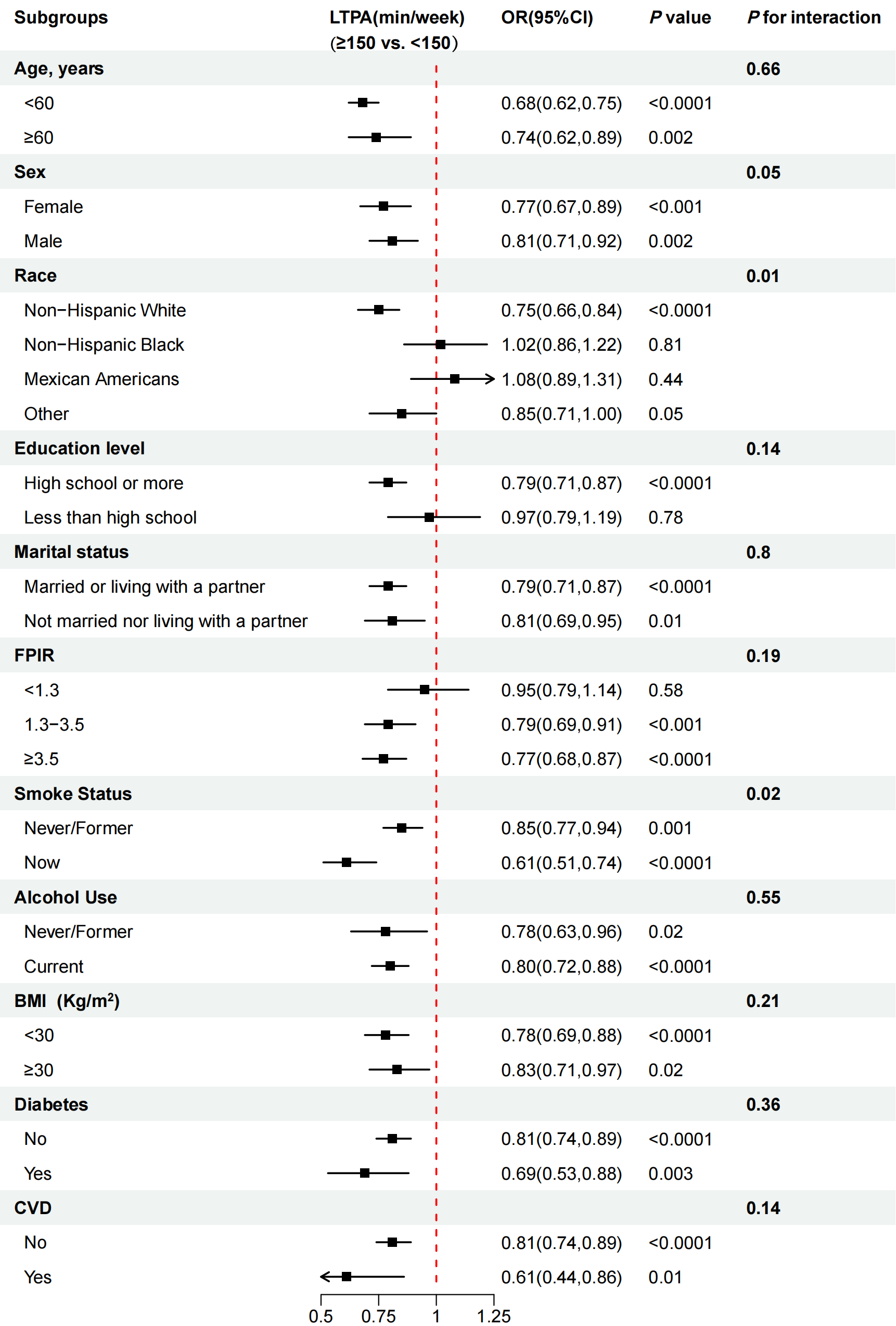

Supplement: Supplementary file 3 — Figure S3: Subgroup analyses of the association between LTPA and hypertension. [file FSN3-13-e71245-s003.tif]
