# Supplementary material for: Dietary Index for Gut Microbiota and Leisure Time Physical Activity: The Potential Combined Protective Impact on Hypertension Risk
Source: Food Sci Nutr. 2025 Nov 27;13(12):e71245. doi: 10.1002/fsn3.71245 (PMC12661085; doi:10.1002/fsn3.71245)
Supplement: Supplementary file 4 — Table S1: Association between DI‐GM and hypertension among the NHANES 2007–2020 participants after multiple imputation. [file FSN3-13-e71245-s001.docx]

**Supplementary Table 1 Association between DI-GM and hypertension among the NHANES 2007–2020 participants after multiple imputation**

| **Characteristics** | **Crude model** | | **Model 1** | | **Model 2** | |
| --- | --- | --- | --- | --- | --- | --- |
|  | **OR (95% CI)** | ***P* value** | **OR (95% CI)** | ***P* value** | **OR (95% CI)** | ***P* value** |
| **DI-GM scores** | 0.97(0.96, 0.99) | <0.0001 | 0.93(0.92, 0.94) | <0.0001 | 0.96(0.94, 0.97) | <0.0001 |
| **DI-GM group** |  |  |  |  |  |  |
| **0-3** | Reference |  | Reference |  | Reference |  |
| **4** | 0.92(0.87, 0.97) | 0.004 | 0.91(0.85, 0.97) | 0.005 | 0.95(0.89, 1.02) | 0.151 |
| **5** | 0.9(0.85, 0.96) | 0.001 | 0.84(0.78, 0.9) | <0.0001 | 0.91(0.84, 0.98) | 0.009 |
| **≥6** | 0.9(0.85, 0.95) | <0.0001 | 0.74(0.69, 0.79) | <0.0001 | 0.84(0.78, 0.9) | <0.0001 |
| ***P* for trend** |  | <0.0001 |  | <0.0001 |  | <0.0001 |
| **BGMS** | 0.94(0.93, 0.96) | <0.0001 | 0.93(0.91, 0.95) | <0.0001 | 0.94(0.92, 0.96) | <0.0001 |
| **UGBS** | 1.03(1.01, 1.05) | 0.003 | 0.95(0.93, 1.04) | 0.274 | 0.99(0.96, 1.01) | 0.349  0.349 |

The crude model was not adjusted for any covariates. Model 1 = age, sex, and race/ethnicity. Model 2 = Model 1 + (education level, marital status, FPIR, smoking status, alcohol drinking status, total physical activity, CVD, diabetes, eGFR, BMI, and energy intake). Abbreviations: BMI, body mass index; FPIR, family poverty income ratio; eGFR, estimated glomerular filtration rate; CVD, cardiovascular disease; CI, confidence interval; OR, odds ratio; DI-GM, dietary index for gut microbiota; UGMS, unfavorable to gut microbiota score; BGMS, beneficial to gut microbiota score.
